# Surgical management of isolated caecal varices presenting with massive lower gastrointestinal haemorrhage: a case report and review of the literature

**DOI:** 10.1093/jscr/rjad438

**Published:** 2023-08-04

**Authors:** Wanyang Qian, Benjamin M Mac Curtain, Anand Trivedi

**Affiliations:** Department of Surgery, St John of God Subiaco Hospital, Subiaco, WA 6008, Australia; Department of Surgery, St John of God Subiaco Hospital, Subiaco, WA 6008, Australia; Acute Surgical Unit, Fiona Stanley Hospital, Murdoch, WA 6150, Australia

**Keywords:** caecal varices, haemorrhage, ectopic varices, hemicolectomy

## Abstract

Caecal varices are extremely rare with poorly defined management due to paucity of data. A 52-year-old man was diagnosed with a 3-day history of melena with a background of chronic liver disease and non-steroidal anti-inflammatory use. Investigations revealed anaemia with haemoglobin of 62 g/L, liver function derangement (Gamma-glutamyl transferase 251 U/L, alanine transaminase 40 U/L, bilirubin 84 umol/L, alkaline phosphatase 85 U/L), coagulopathy (International Normalized Ratio 1.6) and acute kidney injury (Creatinine 285 umol/L). Gastroscopy demonstrated no signs of upper gastrointestinal bleeding or portal hypertension. A large volume haematochezia occurred necessitating resuscitation with massive transfusion protocol, and colonoscopy was abandoned in favour of computerized tomography (CT) angiography, which revealed a large varix feeding the caecum. Urgent laparotomy and a right hemicolectomy was performed with application of abdominal vacuum dressing. The hemicolectomy sample was opened on back table demonstrating large caecal varix causing intraluminal bleeding. The patient was stabilized in intensive care, and a further laparotomy was performed 2 days later where an end ileostomy was formed. Caecal varices have been reported in literature with management via trans-jugular intrahepatic portosystemic shunt, endoscopically or conservatively with beta-blockade. Here we present, to the best of the author’s knowledge, the first reported case of successful surgical management of caecal varices.

## INTRODUCTION

It is reported that variceal bleeding can be observed in sites barring the gastro-oesophageal region in 30% of cases [1, 2]. In a series of patients presenting with bleeding varices, 14% were observed with a colonic origin [3]. Of course, the expected presentation rate of caecal varices presenting as acute haemorrhage is expected to be < 14% of cases, and to the best of the author’s knowledge an exact number is not reported in the literature. Isolated colonic varices have been described as the rarest type of presentation of varices [4]. Sole caecal varices are rarely reported, and have been reported to be diagnosed through endoscopy or observed incidentally at appendectomy [5].

Trans-jugular intrahepatic portosystemic shunt (TIPS) have been described in the management of bleeding caecal varices [7, 8], whereas endoscopic and endovascular treatment options have also been described [6, 8]. In review of the literature, one report of isolated caecal varices presenting as an acute bleed was found; however, this was treated with TIPS alone and no surgical option was required [9].

Herein, we detail a case report of a patient presenting with caecal varices as a source of haematochezia. This case adds to the existing literature regarding case reports of caecal varices undergoing surgical repair [10] or right hemi-colectomy [11], as this case, to the best of the author’s knowledge, is the first to detail isolated caecal varices presenting as an acute lower gastrointestinal (GI) haemorrhage, requiring a hemi-colectomy, after which the patient survived.

## CASE REPORT

A 52-year-old male presented to our institution’s emergency department with a three-day history of melena. This is with a background of night sweats and 8 kg unintentional weight loss over the preceding 6 months. His past medical history included excess alcohol intake, chronic liver cirrhosis (Childs Pugh B), asthma and smoking. Further history revealed long term non-steroidal anti-inflammatory use. There were no previous endoscopic investigations or surgical history.

On initial presentation, the patient’s vitals consisted of a blood pressure of 98/44 mmHg and a heart rate of 93 bpm. Focused clinical examination demonstrated hepatomegaly and tenderness on palpation of the right upper quadrant. Guarding was present. Rectal examination demonstrated traces of dark red blood but no melena, with no immediate cause of bleeding identifiable. Phlebotomy revealed an anaemia (haemoglobin 62 g/L) in the setting of liver function derangement (gamma-glutamyl transferase 251 U/L, alanine transaminase 40 U/L, bilirubin 84 umol/L, alkaline phosphatase 85 U/L), coagulopathy (International Normalized Ratio 1.6) and acute kidney injury (creatinine 285 umol/L).

**Figure 1:**
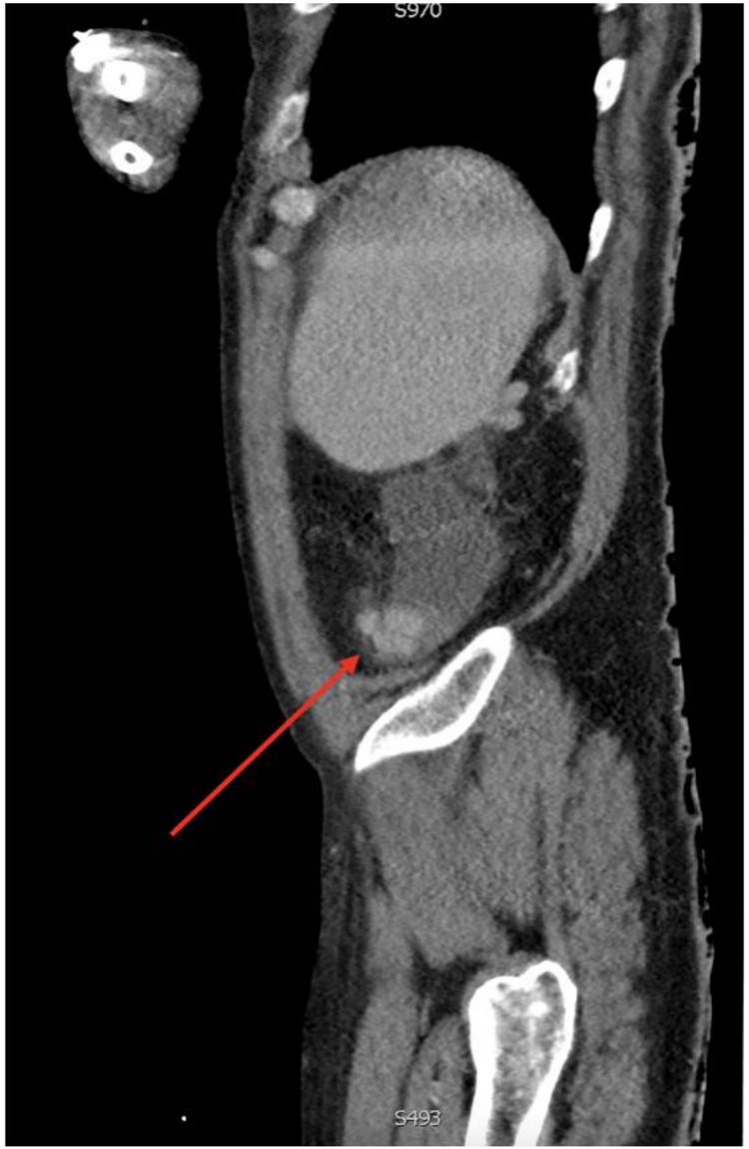
Axial view of computerized tomography imaging in portal venous phase with arrow marking varices at the caecum.

**Figure 2:**
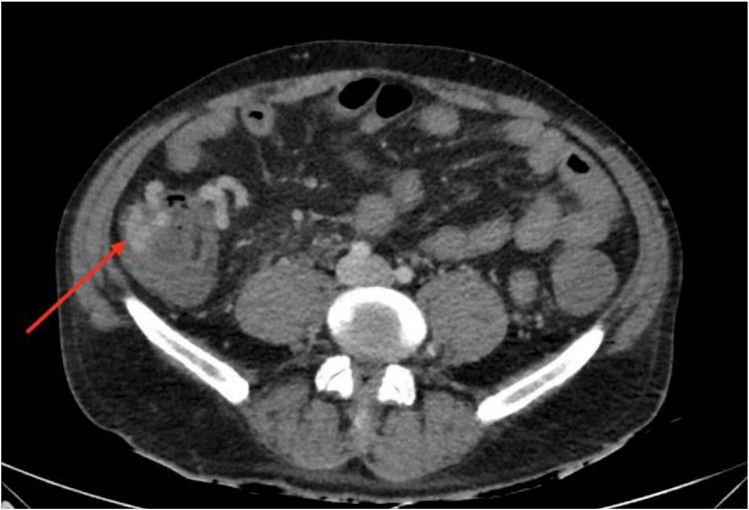
Sagittal view of computerized tomography imaging in portal venous phase with arrow marking varices at the caecum.

On gastroscopy, neither bleeding sources nor features of portal hypertension were identified. Subsequently, a large volume rectal haemorrhage occurred, resulting in severe hypotension and syncope. The patient was resuscitated appropriately and received a massive transfusion protocol. A CT angiogram revealed focal clustered varices travelling retroperitoneally abutting the caecal pole (Fig. 1), which communicated with a large branch of SMV in the right iliac fossa (Fig. 2).

The patient underwent an emergency laparotomy. Intraoperatively large varices supplying the ascending colon and caecum were noted. A right hemicolectomy was performed with stapled bowel ends left *in situ*, and the administration of an abdominal vacuum dressing. The resected bowel specimen was opened on back-table where an isolated dilated varix opening into the caecum was observed to cause intraluminal bleeding. The patient was then transferred to intensive care.

A further laparotomy was performed 2 days later, where extensive oedema in the abdominal wall and bowel was noted. An end ileostomy was performed due to the high likelihood of decompensation and limited patient life expectancy.

The post-operative course was complicated with bacterial peritonitis and hepatorenal syndrome and the patient remained in intensive care for the majority of his inpatient stay. He undergoes follow-up with gastroenterology and renal teams.

## DISCUSSION

Common causes of lower GI bleeding include diverticulosis and neoplasms [12]. Variceal bleeds are an uncommon source of lower GI bleeding and is most commonly caused by portal hypertension [12]. Other aetiologies include biliary atresia, biliary sclerosis, congestive heart failure, and mesenteric vein thrombosis [3]. In relation to cirrhosis and portal hypertension, the vast majority of varices are found in the oesophagus, with 30% originating from extraoesophageal locations [1], also known as ectopic varices. The most common extraoesophageal variceal locations are found in the stomach and rectum [2]. The presence of varices in the caecum is extremely rare and poorly described in the literature.

The diagnosis and management of caecal varices is difficult and has been variably reported largely due to the paucity of data. In the literature, described cases often present with signs of advanced portal hypertension [1, 13]. Diagnosis of caecal varices in literature was conducted through CT angiography or endoscopy, whereas Mikat et al described varices found incidentally on appendicectomy [14]. Cases were managed endovascularly, endoscopically through band ligation, or definitive surgical resection [6, 8, 11]. El-Masry et al. managed non-bleeding caecal varices non-operatively through beta blockade, to good effect [2]. Successful surgical management of isolated caecal varices presenting with acute lower gastrointestinal haemorrhage has not been reported.

Mesocaval/splenorenal shunting and portal vein thrombosis have been shown to be associated with ectopic colonic varices [8, 10]. Their absence in our case on CT angiography excluded TIPS as an acute management option.

In conclusion, we present the first case, to the best of the author’s knowledge, of successful surgical management in massive lower GI bleeding due to isolated caecal varices, in a moribund patient with chronic liver disease, with absence of variceal formation elsewhere. It illustrates the difficulty in diagnosis and the lack of defined management guidelines. This particular diagnosis was testing due to the presence of isolated caecal varices without signs of portal hypertension on gastroscopy. Similarly, the patient underwent no previous abdominal surgeries in the past which has been associated with ectopic varices, and hypothesized to cause unusual collateral formation [9, 10]. The nature of this patient’s unstable presentation, refractory to resuscitation efforts, necessitated a surgical approach. The comorbid nature of the patient in addition to the severity of presentation likely contributed to subpar outcomes of end ileostomy.

## CONFLICT OF INTEREST STATEMENT

The authors have no conflict of interest to declare.

## FUNDING

This work did not receive funding from any source.

## DATA AVAILABILITY

All data is available on request.
